# Radiological Assessment of Frontal Recess Cells and Their Association with Frontal Sinusitis Among Omani Subjects: A Single-Center Computed Tomography-Based Study

**DOI:** 10.3390/medicina62020390

**Published:** 2026-02-17

**Authors:** Srinivasa Rao Sirasanagandla, Noor Fazaldad, Faiza Al Hajri, Tariq Al Habsi, Mohammed Al Washahi, Muataz Al Siyabi, Eiman Al-Ajmi

**Affiliations:** 1 Department of Human and Clinical Anatomy, College of Medicine and Health Sciences, Sultan Qaboos University, Muscat 123, Oman; 2 Diagnsotic Imaging Department, Sohar Hospital, Sohar 311, Oman; noor.fatima39.nm@gmail.com; 3 Department of Radiology and Molecular Imaging, College of Medicine and Health Sciences, Sultan Qaboos University, University Medical City, Muscat 123, Oman; f.alhajri@squ.edu.om; 4 Radiology Residency Program, Oman Medical Specialty Board, Al-Khoudh, Muscat 132, Oman; 124299tariq@gmail.com; 5 Department of Surgery, Ear, Nose and Throat (ENT) Division, College of Medicine and Health Sciences, Sultan Qaboos University, University Medical City, Muscat 123, Oman; malwashahi@squ.edu.om; 6 College of Medicine and Health Sciences, Sultan Qaboos University, Muscat 123, Oman; s129778@student.squ.edu.om

**Keywords:** computed tomography, frontal recess cells, variation, sinusitis, supra bulla cell, supra agger frontal cell, supra orbital ethmoid cell

## Abstract

*Background and Objectives*: Frontal recess cells (FRCs) are key anatomical variants influencing frontal sinus drainage and disease. The International Frontal Sinus Anatomy Classification (IFAC) provides a standardized system for their identification. The baseline data on FRC prevalence and its association with sinusitis in Middle Eastern populations remain limited. This study aimed to assess the prevalence, laterality, and clinical relevance of FRCs in an adult Omani population using IFAC criteria. *Materials and Methods:* Computed tomography (CT) scans of 488 sides from 244 adult Omani patients were retrospectively reviewed to identify the FRCs according to IFAC. A total of 123 patients were found to have sinusitis: bilateral in 37 patients, right-sided in 38 patients, and left-sided in 48 patients. The prevalence of FRCs, along with their laterality and gender differences, was evaluated. Furthermore, the association between FRCs and sinusitis was analyzed using a chi-square test, followed by multivariable logistic regression analysis. *Results*: Among overall subjects (244 patients; 488 sides), agger nasi cells (79.5%, n = 388) and supra bulla cells (64.1%, n = 313) were the most prevalent cell types, whereas frontal septal cells (8.8%, n = 43) were the least common. Among study subjects (n = 244), laterality differences in FRC frequency were observed in the frontal septal cells, with a higher frequency of cells on the right side (*p* = 0.01), while no significant differences were found in other cell types. The presence of supra agger frontal cells (OR = 1.544, 95% CI: 1.02–2.23, *p* = 0.03) was positively correlated with sinusitis, while the presence of supra bulla cells was inversely associated with frontal sinusitis (OR = 0.61, 95% CI: 0.41–0.93, *p* = 0.01). The presence of FRC type was not significantly associated with the degree of sinusitis (*p* > 0.05). Significant gender differences were observed in supra orbital ethmoid cells (*p* = 0.01), with a male predominance. *Conclusions*: This study provides baseline data on the prevalence of FRCs in the Omani population. The presence of supra agger frontal cells appears to be associated with frontal sinusitis. No statistically significant gender- or laterality-associated differences were observed in most FRC types. Preoperative identification of supra agger frontal cells may facilitate effective surgical planning, particularly for endoscopic sinus surgery.

## 1. Introduction

Chronic rhinosinusitis (CRS) is a persistent inflammatory condition of the nasal and paranasal sinuses lasting more than 12 weeks, significantly impacting public health and patients’ quality of life [1,2]. Globally, it affects up to 12% of the population [3], leading to symptoms such as nasal obstruction, facial pressure or pain, hyposmia, and nasal discharge [4]. It is broadly categorized into two phenotypes, with and without nasal polyps, both of which contribute to health-related low quality of life, poor work productivity, sleep disturbances, loss of olfaction and sexual function, and, subsequently, financial and psychosocial burdens [1,4]. In secondary or tertiary care, computed tomography (CT) plays an important role in diagnosis, to monitor the progression of the disease, and to plan the treatment, particularly in cases unresponsive to medical therapy [1]. In CRS patients, CT helps the radiologist in assessing the mucosal thickening or opacification and detecting anatomical variants that may predispose a patient to recurrent disease [2,5]. Moreover, CRS is a multifactorial disease, influenced by environmental exposures such as allergens and smoking, anatomical variants such as septal deviation, microbial factors, immunological factors, and ciliary dysfunction [6,7,8]. The complex nature of the disease necessitates personalized management in CRS patients. In this context, exploring the anatomical variants that influence the etiology of the CRS is clinically relevant.

The frontal sinus and its drainage pathway, particularly the frontal recess, are among the most anatomically complex regions of the paranasal sinuses. Endoscopic sinus surgery is a frequent surgical option in cases of persistent symptoms or failure of medical treatment [9]. The close proximity of the frontal sinus and frontal recess with critical structures, such as the anterior ethmoid artery, anterior skull base, cribriform plate, olfactory apparatus, and medial orbital wall, makes the surgery technically challenging and potentially risky [9,10]. Henceforth, knowledge of these complex structures is clinically important for successful surgical outcomes. Frontal recess cells (FRCs) are ethmoidal air cells that encircle, and may narrow, the frontal sinus drainage pathway, potentially predisposing it to frontal sinusitis [11,12]. Although several classification systems are proposed for FRCs [12,13,14,15], the International Frontal Sinus Anatomy Classification (IFAC), based on three types of cells, is the most widely accepted classification in clinical practice [16]. According to IFAC, the FRCs are classified into agger nasi cell (ANC), supra agger cell (SAC), supra agger frontal cell (SAFC), supra bulla cell (SBC), supra bulla frontal cell (SBFC), supra orbital ethmoid cell (SOEC), and frontal septal cell (FSC). In the existing literature, the reported prevalence rates of FRCs varied across different populations, which could be attributed to differences in imaging protocols, classification systems, and ethnic backgrounds [17,18,19,20,21,22]. Furthermore, sex-based differences in FRC prevalence have been reported in a few studies [17,19]. The presence of a specific type of FRCs may increase the risk of developing frontal sinusitis [17,20].

Prior knowledge about the pneumatization patterns of frontal recess cells and their possible variations is clinically important for safe and effective endoscopic sinus surgery [23]. Misidentification or incomplete removal of these cells may increase the risk of recurrence of the disease [24]. To date, no study has evaluated the prevalence of FRCs or their association with frontal sinusitis in populations from the Arab Gulf countries. Furthermore, existing studies in other populations demonstrate conflicting results regarding this association [17,18,19,20,21,22,25]. Additionally, the influence of FRCs’ presence on the severity of sinusitis has not been studied. Hence, the present study aimed to investigate the prevalence of FRCs, as well as sex- and laterality-associated differences in their occurrence in the Omani population, using the IFAC. The association between FRC occurrence and frontal sinusitis development was also examined.

## 2. Materials and Methods

### 2.1. Study Design and Setting

This retrospective cross-sectional study was conducted at Sultan Qaboos University Hospital, a tertiary care center in Muscat, Oman. CT scans of the paranasal sinuses performed between January 2020 and May 2021 were included. The ethics committee’s approval was obtained for this study from the institutional ethics committee (REF. NO. SQU-EC/323/2023).

### 2.2. Study Population

Omani adults (≥18 years) of either sex who underwent CT of the paranasal sinuses were included in the study. Patients with conditions that are likely to alter frontal recess anatomy, including sinus surgery, maxillofacial fracture, sinonasal malignancy, fungal sinusitis, and congenital anomalies, as well as CT with motion defects, were excluded from the study. A total of 488 sides of paranasal sinus CT images from 244 patients were reviewed to document the presence of FRCs as per the IFAC. All patients underwent CT for various sinonasal complaints, except for eight patients who were scanned for preoperative planning of trans-sphenoidal surgery. Among the study sample, the females represented 57.4%. The included patients’ age range was 18 to 78 years, with a mean age of 39.7 years (standard deviation: 13.5).

### 2.3. CT Acquisition Protocol

CT scans were acquired using a SOMATOM Force CT scanner (Siemens Healthineers, Erlangen, Germany). CT scans of the paranasal sinuses were performed for all patients with imaging parameters including tube voltage of 100 kV, automated exposure control (CareDose) for a tube current modulation, and a slice thickness of 0.6 mm.

### 2.4. Imaging Review and Classification

**Figure 1:**
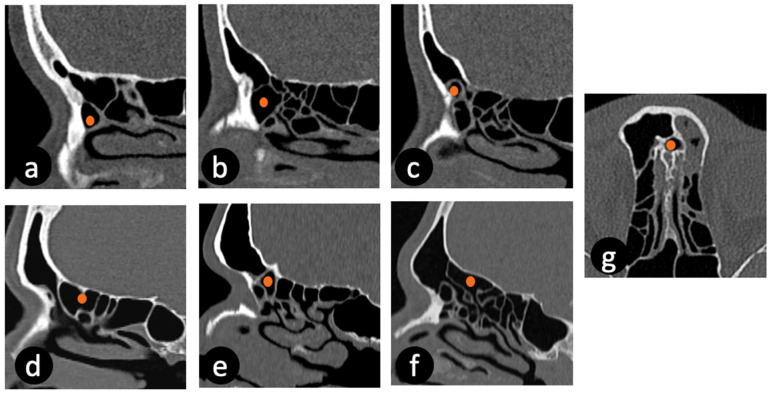
Frontal recess cell types according to the International Frontal Sinus Anatomy Classification on sagittal (**a**–**f**) and axial (**g**) computed tomography images: (**a**) aggar nasi cell, (**b**) supra aggar cell, (**c**) supra aggar frontal cell, (**d**) supra bulla cell, (**e**) supra bulla frontal cell, (**f**) supra orbital ethmoid cell, and (**g**) frontal septal cell type. In each image, the orange dot denotes the position of the specified cell type.

All CT images in axial, coronal, and sagittal planes were reviewed by two radiologists to identify FRCs according to the IFAC (Figure 1). Cells were categorized into anterior, posterior, and medial groups. The frontal sinuses were assessed for radiological evidence of sinusitis. Sinusitis was defined as per the modified Lund–Mackay (LM) scoring of mucosal thickness or opacification by examining the sinuses visually in 3 planes but without volumetric measurement [26]. A mucosal thickness of sinuses less than 3 mm was classified as having no sinusitis. In this scoring system, disease severity is evaluated by using a score ranging from 0 to 5 based on the percentage of mucosal thickening in the opacified sinus, with 0% corresponding to 0 points, <25% to 1 point, 26–50% to 2 points, 51–75% to 3 points, 76–99% to 4 points, and 100% to 5 points. According to the modified LM score, sinusitis was identified in 123 of 244 patients (50.4%): bilateral in 37 patients, right-sided in 38 patients, and left-sided in 48 patients. Any discrepancies in interpretation were resolved by consensus and expert supervision.

### 2.5. Statistical Analysis

All analyses were performed using the Statistical Package for the Social Sciences (SPSS version 26.0, IBM Corp., Armonk, NY, USA). Continuous variables were expressed as mean ± standard deviation (SD), and categorical variables as frequencies and percentages. The chi-square test was used to evaluate the sex differences in FRC occurrence. The McNemar’s test was used to assess any potential laterality differences in the FRC occurrence. Furthermore, the association between the prevalence of FRCs and sinusitis, both the presence and severity of sinusitis, was determined using a chi-square test. Logistic regression analysis was performed to examine the association between frontal recess cell (FRC) types and the presence of frontal sinusitis. The dependent (response) variable was the presence of frontal sinusitis, which was coded as a binary outcome (0 = no sinusitis, 1 = sinusitis). The final multivariable model was constructed using a stepwise backward elimination approach based on the likelihood ratio test. The independent (explanatory) variables included the FRC types classified according to the IFAC system, namely ANC, SAC, SAFC, SBC, SBFC, SOEC, and FSC. Each anatomical variant was treated as a categorical variable and coded dichotomously (0 = absent, 1 = present). For each variable, the absence of the cell was used as the reference category. A *p*-value < 0.05 was considered statistically significant.

## 3. Results

### 3.1. Frontal Recess Cell Prevalence

The FRCs were grouped according to the IFAC. Anterior cells are those that lie anterior to or immediately beneath the frontal ostium and tend to push the frontal sinus drainage pathway medially, posteriorly, or posteromedially; this group includes ANC, SAC, and SAFC. Posterior cells are located posterior to the frontal ostium/frontal recess; this group comprises the SBC, SBFC, and SOEC. The single medial cell in the IFAC is the frontal FSC, which is situated medial to the frontal recess/ostium near the vertical lamella of the middle turbinate and may extend into the frontal sinus. Table 1 represents the overall prevalence of FRCs among study subjects (n = 488 sides), along with the distribution of cells on the right side and left side. The ANC type was found to be the most prevalent cell among the anterior group, with a frequency of 79.5% (n = 388) of the total, followed by SAFC (n = 183, 37.5%) and SAC (n = 69, 14.1%), respectively. Among the posterior group, SBC was the most prevalent with a frequency of 64.1% (n = 313), followed by SOEC (n = 59, 12.1%) and SBFC (n = 46, 9.4%), respectively. FSC is the only medial cell with the least frequency (n = 43, 8.8%) when compared to other groups.

### 3.2. Gender Differences in Frontal Recess Cells Occurrence

In relation to the FRC distribution based on gender, similar frequencies were observed in both genders for all cell types. Among study subjects (n = 488 sides), the distribution of FRC types by gender revealed no statistically significant difference for most cells, except for SOEC (Table 2). Among the anterior group of cells, ANC was the most common, identified in 57.5% of females and 42.5% of males. Within the posterior group cells, SBC was the most prevalent, being present in 58.8% of females and 41.2% of males. For the medial group of cells, FSC was detected in 51.2% of females and 48.8% of males. Significant gender differences were observed in SOEC with a *p*-value of 0.01 (males: 57.6% and females: 42.4%; Table 2).

### 3.3. Effect of Laterality on Frontal Recess Cells Occurrence

The McNemar’s test was used to assess any potential laterality differences in the FRC occurrence among overall study subjects (n = 244 subjects), and the results are presented in Table 3. The analysis showed significant differences between left and right sides for only the FSC type (*p* = 0.01). The frontal septal cell was more frequently detected on the right side (n = 25; 10.2%) than on the left side (n = 10; 4.1%). Among other cell types, including ANC (*p* = 0.90), SAC (*p* = 0.78), SAFC (*p* = 0.83), SBC (*p* = 0.81), SBFC (*p* = 0.58), and SOEC (*p* = 0.52), no significant differences between the sides were detected.

### 3.4. Association Between Frontal Sinusitis and Frontal Recess Cell Frequency

The analysis examined the association between different FRC types and the presence of frontal sinusitis. Overall, most FRC variants did not demonstrate a statistically significant relationship with frontal sinusitis. The SAFC type demonstrated an association with frontal sinusitis (*p* = 0.05), where the prevalence of sinusitis was higher in cases where SAFC was present. A significant association was also observed in the SBC type (*p* = 0.01). Patients without SBCs showed a higher prevalence of frontal sinusitis compared to those with SBCs, suggesting that the presence of SBCs might be less likely to be associated with frontal sinusitis. All other FRC types (ANC, SAC, SBFC, SOEC, and FSC) showed no statistically significant associations with frontal sinusitis (*p* > 0.05), indicating that their presence alone may not substantially influence the occurrence of frontal sinusitis (Table 4). After applying multivariable logistic regression analysis to assess the robustness of the association between the presence of FRC types and the occurrence of frontal sinusitis, two cells were found to be significantly associated with sinusitis (SAFC and SBC). The presence of SAFC was positively correlated with sinusitis, with an odds ratio (OR) of 1.544 (95% CI: 1.02–2.23, *p* = 0.03), indicating that its presence may increase the likelihood of sinusitis. In contrast, the presence of SBCs was inversely associated with frontal sinusitis (OR = 0.61, 95% CI: 0.41–0.93, *p* = 0.01) (Table 5).

To evaluate the relationship between FRC type and the degree of frontal sinusitis, the severity of frontal sinusitis was analyzed in relation to the distribution of cells on the ipsilateral side. On the right side, no role for cell type was noted in association with the right frontal sinusitis severity (*p* > 0.05) (Supplementary Table S1). Similarly, on the left side, no significant association was found between the frequency of cell type and the frontal sinusitis severity (*p* > 0.05) (Supplementary Table S2). However, it is worth mentioning that, given the low representation of moderate and severe sinusitis in association with most cell groups, no firm conclusions can be drawn from the available results.

## 4. Discussion

The present study represents the first CT-based evaluation of frontal recess anatomy using the IFAC in an Omani population. The study provides comprehensive data on the prevalence of FRCs, demographic influences on the occurrence of cells, and their relationship with frontal sinusitis. Before the IFAC adoption, Bent and Kuhn classifications were proposed to describe frontal recess anatomy based on four cell types [12,13]. Although these classifications slightly improved the understanding of cells, they had many limitations in describing the detailed relationships of cells, which are crucial for planning and successful surgical outcomes. Subsequently, the IFAC was proposed to provide standardized, anatomy-based terminology that enables reproducible assessments of FRCs and facilitates reliable surgical interventions in this region [16].

In recent years, due to the complexity of frontal recess anatomy, potential variations, and association with sinusitis, various IFAC-based studies have reported the FRC’s prevalence across many populations worldwide [17,18,19,20,21,22,27,28,29,30,31,32,33,34,35,36]. In reported data, inter-study differences exist among the three groups of FRCs, reflecting substantial geographic variations in their occurrence [17,18]. In most of the studies, the reported frequencies of ANC are higher than 90%, and this high frequency is due to its consistent position and relatively easy identification [17,18,19,20,21,22,27,28,29,30,31,32,33,34,35,36]. In the Omani population, the ANC was observed in 79.5%. Similarly, relatively lower frequencies of ANC were reported in two different studies of Turkish populations (86.3% and 88%) and in the Caucasian population (86.9%) [5,33,35]. In this study, the SACs were identified in 14.1% of cases, which is lower than the reported range of 16.3% to 57% [17,28]. The other anterior group cell, the SAFC, was identified with a frequency of 37.5%, which is comparable to two studies from other Asian countries, India (37.8%) and Malaysia (36%) [20,34]. The frequencies of SBCs were reported with a wide range of 36.1% to 88.8% among different populations [19,24]. In Omani subjects, these cells occurred in 64.1% of the frequency, which is close to the values reported in Taiwanese, Malaysian, Indian, and Egyptian populations [18,20,32,34]. In the reported studies, the presence of SBFCs and SOECs was highly variable; in Omani subjects, their frequencies were less frequent than in the few published studies; however, they were within the range of 4.3% to 58.5% and 5.8% to 51.1%, respectively [5,28,33,34]. Finally, the FSC was the least frequent in this study sample (8.8%), which is close to the value reported in the Malaysian population [20]. Reported FSC prevalence in IFAC studies is highly variable, ranging from 3% to 33% [17,18,19,20,21,22,27,28,29,30,31,32,33,34,35,36]. In a Turkish pediatric study, the absence of FSC was reported [33]. Some of the IFAC studies did not evaluate the FRCs’ association with the development of sinusitis [5,24,27,28,29,31,35].

Overall, in the Omani population, the frequencies of most of the cell types differ from those of other populations, although some of the cell-type frequencies are close to values reported in Malaysia and India and, to a certain extent, with the Turkish and Egyptian populations. Based on these findings, it can be hypothesized that variation in FRC frequencies among different populations could be attributed to racial differences in FRC occurrences. In support of this, in a study, SAC was most prevalent in the white population, while SOEC was most prevalent in the Asian population [17]. However, further studies are needed to evaluate developmental and/or genetic influences on frontal recess cell occurrence.

In the present study, laterality differences in FRC frequency were observed in the FSC type, with a higher frequency of cells on the right side. No significant differences were found in other cell types. Laterality differences were not evaluated in previous studies, except in two studies: one from Taiwan and the other from India [18,34]. However, in these two studies, no statistically significant laterality differences were observed [18,34]. The sexual dimorphism of FRC occurrence has not been well studied in the reported studies. In this study, only the SOEC demonstrated a sex difference, occurring more often in males. Similar to these results, Howser et al. have demonstrated a male predominance for some less common FRCs, including SAFC and SOEC [17]. Overall, the current evidence indicates a possible male predominance for the SOEC occurrence. The influence of sex hormones on craniofacial structure development could be one of the possible explanations for sex-based differences [37].

Discrepancy exists in the literature with regard to the role of FRCs in the development of sinusitis. The reported data on the association between FRCs and sinusitis are presented in Table 6. Previously, a few studies demonstrated the significant association between the presence of SOEC, SBFC, FSC, SAC, and SAFC and the increased risk of developing sinusitis [17,20,30,32,34,36]. In the present study, the presence of SAFC was associated with increased odds, indicating a potential anatomical influence that warrants further investigation with a larger sample size. The position of SAFC and SOEC may encroach on the infundibulum, causing frontal sinus drainage pathway obstruction, which could be the causative factor for the increased risk of developing sinusitis. In contrast, the inverse association was observed for SBC, which is less frequently described in the literature. It has been reported that the SBC pneumatization pattern has a valid effect on the surrounding anatomical structures, including the frontal sinus and its drainage pathway [38]. Four types of SBC pneumatization have been described, but such an additional assessment was not included in IFAC [38]. Given the current results of the present study, SBC pneumatization could be an explanation for why the presence of SBCs was less likely associated with frontal sinusitis. However, such a broad conclusion cannot be generalized due to the limited sample size, and further assessment of the pneumatization pattern was beyond the scope of this study. Yet, this sheds light on a research gap that could be explored in a future study. In this study, the degree of sinusitis showed no association with the presence or type of FRCs, suggesting that FRC types have minimal influence on disease severity. Across IFAC studies, results regarding associations with sinusitis vary widely, likely reflecting differences in study design, sample size, CT imaging protocols, and diagnostic approaches [17,18,19,20,21,22,32,36]. Henceforth, future studies on large sample sizes are warranted to confirm such unique observations.

In the present study, the negative association of SBCs with frontal sinusitis suggests that not all anatomical variations increase disease risk. This emphasizes that clinical decision-making should not rely solely on the presence or absence of individual cells but rather on a comprehensive three-dimensional assessment of the frontal recess, including drainage pathways and the spatial relationships of coexisting cells. A thorough understanding of FRC patterns and drainage pathways is clinically important for successful surgical outcomes. During endoscopic sinus surgery, identifying FRC variants is important to safely remove the cells obstructing the frontal sinus drainage pathway, avoid damage to surrounding structures, and prevent complications or disease recurrence. For example, in cases of the presence of a large SAFC, it is essential to remove the bulla and cells located above it for the safe exposure of the frontal recess and sinus drainage without damaging nearby orbital structures [25]. Therefore, incorporation of IFAC into routine radiological reporting enhances surgical safety and effectiveness by allowing precise identification of key anatomic variants [10,16].

The present study has the following limitations. As the study was conducted at a single tertiary care center, the findings may not be generalized to the broader Omani or Gulf population, though it is a tertiary care referral center with samples from different parts of Oman. Moreover, the inclusion of patients who are primarily presented with sinonasal complaints might have resulted in selection bias. Formal reliability testing was not performed, although the inter-observer variability was reduced through consensus and expert supervision.

## 5. Conclusions

Overall, in the Omani population, the frequencies of most of the cell types differ significantly from those of other populations. The results of the present study will add to the pooled data on FRCs in the existing literature. However, some of the cell-type frequencies are close to values reported in studies of Asian populations. Most of the FRC type frequencies were not associated with either gender or laterality. Significant gender differences were observed in SOEC, with a male predominance. Laterality differences were observed in FSC type with right-sided dominance. The presence of SAFC appears to increase the susceptibility to developing frontal sinusitis. On the other hand, the presence of SBCs was inversely associated with sinusitis. A future study with a large sample size is warranted to further explore such unique findings for SBCs. Preoperative identification of specific FRC type may facilitate effective surgical planning, particularly during endoscopic sinus surgery in patients with sinusitis.

## Figures and Tables

**Table 1:** Prevalence of frontal recess cells among the study subjects (n = 488 sides).

Frontal Recess Cells	Right Side	Left Side	Total (n)	Percentage (%)
ANC	193	195	388	79.5
SAC	33	36	69	14.1
SAFC	90	93	183	37.5
SBC	155	158	313	64.1
SBFC	21	25	46	9.4
SOEC	27	32	59	12.1
FSC	29	14	43	8.8

ANC: Agger nasi cell; FSC: Frontal septal cell; SAC: Supra agger cell; SAFC: Supra agger frontal cell; SBC: Supra bulla cell; SBFC: Supra bulla frontal cell; SOEC: Supra orbital ethmoid cell.

**Table 2:** Gender differences in the frequency of frontal recess cell occurrence (n = 488 sides).

Frontal Recess Cells Type		Gender			
		Male	Female	Total	
		Count n (%)	Count n (%)	Count n (%)	*p*-Value
ANC	Absent	43 (43)	57 (57.0)	100 (20)	1
	Present	165 (42.5)	223 (57.5)	388 (80)	
SAC	Absent	182 (43.4)	237 (56.6)	419 (86)	0.44
	Present	26 (37.7)	43 (62.3)	69 (14)	
SAFC	Absent	124 (40.7)	181 (59.3)	305 (63)	0.29
	Present	84 (45.9)	99 (54.1)	183 (38)	
SBC	Absent	79 (45.1)	96 (54.9)	175 (36)	0.45
	Present	129 (41.2)	184 (58.8)	313 (64)	
SBFC	Absent	188 (42.5)	254 (57.5)	442 (91)	1
	Present	20 (43.5)	26 (56.5)	46 (9)	
SOEC	Absent	174 (40.6)	255 (59.4)	429 (88)	0.01 *
	Present	34 (57.6)	25 (42.4)	59 (12)	
FSC	Absent	187 (42.0)	258 (58.0)	445 (91)	0.48
	Present	21 (48.8)	22 (51.2)	43 (9)	

ANC: Agger nasi cell; FSC: Frontal septal cell; SAC: Supra agger cell; SAFC: Supra agger frontal cell; SBC: Supra bulla cell; SBFC: Supra bulla frontal cell; SOEC: Supra orbital ethmoid cell. * *p*-value < 0.05; chi-square test.

**Table 3:** Laterality differences in frequency of frontal recess cell occurrence (n = 244 subjects).

Frontal Recess Cells Type	Side		Left			McNemar χ^2^ Value	*p*-Value
		Status	Absent Count n (%)	Present Count n (%)	Total Count n (%)		
ANC	Right	Absent	13 (5.3)	38 (15.6)	51 (20.9)	0.01	0.90
		Present	36 (14.8)	157 (64.3)	193 (79.1)		
		Total	49 (20.1)	195 (79.9)	244 (100)		
SAC	Right	Absent	183 (75)	28 (11.5)	211 (86.5)	0.07	0.78
		Present	25 (10.2)	8 (3.3)	33 (13.5)		
		Total	208 (85.2)	36 (14.8)	244 (100)		
SAFC	Right	Absent	104 (42.6)	50 (20.5)	154 (63.1)	0.04	0.83
		Present	47 (19.3)	43 (17.6)	90 (36.9)		
		Total	151 (61.9)	93 (38.1)	244 (100)		
SBC	Right	Absent	53 (21.7)	36 (14.8)	89 (36.5)	0.05	0.81
		Present	33 (13.5)	122 (50.0)	155 (63.5)		
		Total	86 (35.2)	158 (64.8)	244 (100)		
SBFC	Right	Absent	206 (84.4)	17 (7.0)	223 (91.4)	0.30	0.58
		Present	13 (5.3)	8 (3.3)	21 (8.6)		
		Total	219 (89.8)	25 (10.2)	244 (100)		
SOEC	Right	Absent	195 (79.9)	22 (9.0)	217 (88.9)	0.41	0.52
		Present	17 (7.0)	10 (4.1)	27 (11.1)		
		Total	212 (86.9)	32 (13.1)	244 (100)		
FSC	Right	Absent	205 (84.0)	10 (4.1)	215 (88.1)	5.60	0.01 *
		Present	25 (10.2)	4 (1.6)	29 (11.9)		
		Total	230 (94.3)	14 (5.7)	244 (100)		

A * *p*-value < 0.05 indicates a statistically significant difference between the left- and right-sided cell presence. ANC: Agger nasi cell; FSC: Frontal septal cell; SAC: Supra agger cell; SAFC: Supra agger frontal cell; SBC: Supra bulla cell; SBFC: Supra bulla frontal cell; SOEC: Supra orbital ethmoid cell; McNemar test.

**Table 4:** Association between frontal recess cells and frontal sinusitis (n = 488 sides).

Frontal Recess Cells Type		Frontal Sinus			
		No Sinusitis	With Sinusitis	Total	
		Count n (%)	Count n (%)	Count n (%)	*p*-Value
ANC	Absent	68 (68.0)	32 (32)	100 (20.5)	0.94
	Present	260 (67.0)	128 (33.0)	388 (79.5)	
SAC	Absent	277 (66.1)	142 (33.9)	419 (85.9)	0.25
	Present	51 (73.9)	18 (26.1)	69 (14.1)	
SAFC	Absent	215 (70.5)	90 (29.5)	305 (62.5)	0.05 *
	Present	113 (61.7)	70 (38.3)	183 (37.5)	
SBC	Absent	105 (60.0)	70 (40.0)	175 (35.9)	0.01 *
	Present	223 (71.2)	90 (28.8)	313 (64.1)	
SBFC	Absent	296 (67.0)	146 (33.0)	442 (90.6)	0.84
	Present	32 (69.6)	14 (30.4)	46 (9.4)	
SOEC	Absent	294 (68.5)	135 (31.5)	429 (87.9)	0.12
	Present	34 (57.6)	25 (42.4)	59 (12.1)	
FSC	Absent	298 (67.0)	147 (33.0)	445 (91.2)	0.83
	Present	30 (69.8)	13 (30.2)	43 (8.8)	

ANC: Agger nasi cell; FSC: Frontal septal cell; SAC: Supra agger cell; SAFC: Supra agger frontal cell; SBC: Supra bulla cell; SBFC: Supra bulla frontal cell; SOEC: Supra orbital ethmoid cell. * *p*-value < 0.05; chi-square test.

**Table 5:** Association between frontal recess cells and frontal sinusitis using multivariable regression analysis.

Variable (Frontal Recess Cell Type)	B	S.E.	Wald Statistics	Difference	Sig.	Exp (B)	95% C.I. Lower	95% C.I. Upper
ANC	0.149	0.252	0.351	1	0.55	1.161	0.709	1.901
SAC	−0.261	0.305	0.731	1	0.39	0.770	0.424	1.401
SAFC	0.434	0.207	4.386	1	0.03 *	1.544	1.028	2.319
SBC	−0.483	0.207	5.473	1	0.01 *	0.617	0.411	0.925
SBFC	−0.118	0.343	0.119	1	0.73	0.888	0.454	1.739
SOEC	0.336	0.295	1.296	1	0.25	1.400	0.784	2.498
FSC	−0.076	0.356	0.045	1	0.83	0.927	0.461	1.863

ANC: Agger nasi cell; FSC: Frontal septal cell; SAC: Supra agger cell; SAFC: Supra agger frontal cell; SBC: Supra bulla cell; SBFC: Supra bulla frontal cell; SOEC: Supra orbital ethmoid cell; B: coefficients beta; S.E.: standard errors. Logistic regression analysis. * *p*-value < 0.05.

**Table 6:** The association between frontal recess cells and sinusitis from different studies.

Study	Year	Frontal Recess Cell Variation Association with Sinusitis	Sinus Cell Type Associated with Sinusitis
Seth et al. [19]	2020	No	N/A
Pham et al. [30]	2021	Yes	SAFC, SBFC (±SAC in isolated FS)
Fawzi et al. [20]	2022	Yes	SOEC, FSC
Nofal & El-Anwar [21]	2022	No	N/A
Oraby et al. [32]	2023	Yes	SAC, SOEC
Howser et al. [17]	2023	Yes	SOEC
Nair et al. [34]	2024	Yes	SBFC
Loburets et al. [22]	2024	No	N/A
Kho et al. [36]	2025	Yes	SAFC, SBFC
Chang et al. [18]	2025	No	N/A
Present study	2026	Yes	SAFC

SAC: Supra agger cell; SAFC: Supra agger frontal cell; SBFC: Supra bulla frontal cell; SOEC: Supra orbital ethmoid cell; FSC: Frontal septal cell; NA: not available.

## Data Availability

The data is available upon request from the corresponding authors.

## Supplementary Materials

The following supporting information can be downloaded at: https://www.mdpi.com/article/10.3390/medicina62020390/s1; Table S1: Association between right-sided cell distribution and the severity of right frontal sinusitis based on the Modified Lund-Mackay score (244 patients); Table S2: Association between left-sided cell distribution and the severity of left frontal sinusitis based on the Modified Lund-Mackay score (244 patients).

## Abbreviations

CT	Computed tomography
CRS	Chronic rhinosinusitis
FRC	Frontal recess cell
ANC	Agger nasi cell
FSC	Frontal septal cell
SAC	Supra agger cell
SAFC	Supra agger frontal cell
SBC	Supra bulla cell
SBFC	Supra bulla frontal cell
SOEC	Supra orbital ethmoid cell
IFAC	International Frontal Sinus Anatomy Classification
