# Supplementary material for: Radiological Assessment of Frontal Recess Cells and Their Association with Frontal Sinusitis Among Omani Subjects: A Single-Center Computed Tomography-Based Study
Source: Medicina (Kaunas). 2026 Feb 17;62(2):390. doi: 10.3390/medicina62020390 (PMC12943481; doi:10.3390/medicina62020390)
Supplement: Supplementary file 1 [file medicina-62-00390-s001.zip › medicina-4020335-supplementary.pdf]

Supplementary Table S1: Association between right-sided cell distribution and the severity of right frontal sinusitis based on the Modified Lund-Mackay score (244 patients).

FRC TYPE		The severity degree of right frontal sinusitis														
		0 %		<25 %		26-50 %		51-75 %		76-99 %		100 %		Total		
		Count	Row N %	Count	Row N %	Count	Row N %	Count	Row N %	Count	Row N %	Count	Row N %	Count	%	p-value
ANC	Absent	38	74.5%	6	11.8%	1	2.0%	1	2.0%	1	2.0%	4	7.8%	51	20.9%	0.817
	Present	131	67.9%	25	13.0%	13	6.7%	6	3.1%	5	2.6%	13	6.7%	193	79.1%	
SAC	Absent	145	68.7%	28	13.3%	10	4.7%	7	3.3%	6	2.8%	15	7.1%	211	86.5%	0.387
	Present	24	72.7%	3	9.1%	4	12.1%	0	0.0%	0	0.0%	2	6.1%	33	13.5%	
SAFC	Absent	113	73.4%	15	9.7%	8	5.2%	5	3.2%	4	2.6%	9	5.8%	154	63.1%	0.401
	Present	56	62.2%	16	17.8%	6	6.7%	2	2.2%	2	2.2%	8	8.9%	90	36.9%	
SBC	Absent	55	61.8%	13	14.6%	8	9.0%	1	1.1%	4	4.5%	8	9.0%	89	36.5%	0.118
	Present	114	73.5%	18	11.6%	6	3.9%	6	3.9%	2	1.3%	9	5.8%	155	63.5%	
SBFC	Absent	154	69.1%	27	12.1%	13	5.8%	6	2.7%	6	2.7%	17	7.6%	223	91.4%	0.664
	Present	15	71.4%	4	19.0%	1	4.8%	1	4.8%	0	0.0%	0	0.0%	21	8.6%	
SOEC	Absent	154	71.0%	23	10.6%	13	6.0%	6	2.8%	5	2.3%	16	7.4%	217	88.9%	0.125
	Present	15	55.6%	8	29.6%	1	3.7%	1	3.7%	1	3.7%	1	3.7%	27	11.1%	
FSC	Absent	147	68.4%	27	12.6%	13	6.0%	7	3.3%	5	2.3%	16	7.4%	215	88.1%	0.821
	Present	22	75.9%	4	13.8%	1	3.4%	0	0.0%	1	3.4%	1	3.4%	29	11.9%	

ANC: Agger Nasi Cell; FSC: Frontal Septal Cell; SAC: Supra Agger Cell; SAFC: Supra Agger Frontal Cell; SBC: Supra Bulla Cell; SBFC: Supra Bulla Frontal Cell; SOEC: Supra Orbital Ethmoid Cell. * p-value < 0.05; Chi-square test.

Supplementary Table S2: Association between left-sided cell distribution and the severity of left frontal sinusitis based on the Modified Lund-Mackay score (244 patients).

FRC Type		The severity degree of left frontal sinusitis														
		0 %		<25 %		26-50 %		51-75 %		76-99 %		100 %		Total		
		Count	%	Count	%	Count	%	Count	%	Count	%	Count	%	Count	%	p-value
ANC	Absent	32	65.3%	7	14.3%	3	6.1%	3	6.1%	2	4.1%	2	4.1%	49	20.1%	0.56
	Present	137	70.3%	24	12.3%	11	5.6%	4	2.1%	4	2.1%	17	7.7%	195	79.9%	
SAC	Absent	138	66.3%	28	13.5%	13	6.3%	7	3.4%	5	2.4%	17	8.2%	208	85.2%	0.207
	Present	31	86.1%	3	8.3%	1	2.8%	0	0.0%	1	2.8%	0	0.0%	36	14.8%	
SAFC	Absent	109	72.2%	18	11.9%	8	5.3%	4	2.6%	4	2.6%	8	5.3%	151	61.9%	0.773
	Present	60	64.5%	13	14.0%	6	6.5%	3	3.2%	2	2.2%	9	9.7%	93	38.1%	
SBC	Absent	52	60.5%	14	16.3%	8	9.3%	2	2.3%	3	3.5%	7	8.1%	86	35.2%	0.241
	Present	117	74.1%	17	10.8%	6	3.8%	5	3.2%	3	1.9%	10	6.3%	158	64.8%	
SBFC	Absent	150	68.5%	29	13.2%	11	5.0%	7	3.2%	6	2.7%	16	7.3%	219	89.8%	0.492
	Present	19	76.0%	2	8.0%	3	12.0%	0	0.0%	0	0.0%	1	4.0%	25	10.2%	
SOEC	Absent	150	70.8%	24	11.3%	12	5.7%	7	3.3%	4	1.9%	15	7.1%	212	86.9%	0.286
	Present	19	59.4%	7	21.9%	2	6.3%	0	0.0%	2	6.3%	2	6.3%	32	13.1%	
FSC	Absent	159	69.1%	30	13.0%	14	6.1%	7	3.0%	6	2.6%	14	6.1%	230	94.3%	0.263
	Present	10	71.4%	1	7.1%	0	0.0%	0	0.0%	0	0.0%	3	21.4%	14	5.7%	

ANC: Agger Nasi Cell; FSC: Frontal Septal Cell; SAC: Supra Agger Cell; SAFC: Supra Agger Frontal Cell; SBC: Supra Bulla Cell; SBFC: Supra Bulla Frontal Cell; SOEC: Supra Orbital Ethmoid Cell. * *p*-value < 0.05; Chi-square test.
